# In silico Analysis of Two Novel Variants in the Pyruvate Carboxylase (PC) Gene Associated with the Severe Form of PC Deficiency

**DOI:** 10.61186/ibj.27.5.307

**Published:** 2023-06-19

**Authors:** Fereshteh Maryami, Elham Rismani, Elham Davoudi-Dehaghani, Nasrin Khalesi, Saeed Talebi, Reza Mahdian, Sirous Zeinali

**Affiliations:** 1 Department of Molecular Medicine, Biotechnology Research Center, Pasteur Institute of Iran, Pasteur St., Tehran, Iran;; 2 Department of Pediatrics and Neonatal Intensive Care Unit, Ali-Asghar Children’s Hospital, Iran University of Medical Sciences, Tehran, Iran;; 3 Department of Medical Genetics and Molecular Biology, Faculty of Medicine, Iran University of Medical Sciences (IUMS), Tehran, Iran;; 4 Kawsar Human Genetics Research Center, Tehran, Iran

**Keywords:** Pyruvate carboxylase, Pyruvate carboxylase deficiency disease, Metabolic diseases, Exome sequencing

## Abstract

**Background:**

Inborne errors of metabolism are a common cause of neonatal death. This study evaluated the acute early-onset metabolic derangement and death in two unrelated neonates.

**Methods:**

WES, Sanger sequencing, homology modeling, and in silico bioinformatics analysis were employed to assess the effects of variants on protein structure and function.

**Results:**

WES revealed a novel homozygous variant, p.G303Afs*40 and p.R156P, in the *PC* gene of each neonate, which both were confirmed by Sanger sequencing. Based on the ACMG guidelines, the p.G303Afs*40 was likely pathogenic, and the p.R156P was a VUS. Nevertheless, a known variant at position 156, the p.R156Q, was also a VUS. Protein secondary structure prediction showed changes in p.R156P and p.R156Q variants compared to the wild-type protein. However, p.G303Afs*40 depicted significant changes at C-terminal. Furthermore, comparing the interaction of wild-type and variant proteins with the ATP ligand during simulations, revealed a decreased affinity to the ATP in all the variants. Moreover, analysis of SNP impacts on PC protein using Polyphen-2, SNAP2, FATHMM, and SNPs&GO servers predicted both R156P and R156Q as damaging variants. Likewise, free energy calculations demonstrated the destabilizing effect of both variants on PC.

**Conclusion:**

This study confirmed the pathogenicity of both variants and suggested them as a cause of type B PCD. The results of this study would provide the family with prenatal diagnosis and expand the variant spectrum in the *PC *gene,which is beneficial for geneticists and endocrinologists.

## INTRODUCTION

The *PC* gene (NG_008319.1), of 109,964 bp in length, is located at chromosome 11q13.2 and has three tissue-specific mitochondrial transcripts that encode the PC (EC 6.4.1.1) protein. The PC enzyme has 1,178 amino acids and contains three functional domains: biotin carboxylation, trans-carboxylation, and biotin carboxyl carrier^[^^1^^]^. This enzyme is a homotetramer consisting of four subunits, each subunit has a covalently attached biotin molecule and the binding sites for pyruvate, ATP, acetyl-CoA, and HCO_3_. PC facilitates a two-step reaction in the mitochondrial matrix. The first step is the ATP-dependent carboxylation of the biotin, which covalently binds to the BCCP, and the second step is the transfer of carboxyl group to pyruvate by biotin in the CT domain^[^^2^^,^^3^^]^. The PC enzyme forms oxaloacetate from pyruvate and HCO_3_ in the mitochondrial matrix^[^^4^^,^^5^^]^. Mitochondrial oxaloacetate is a critical substrate for the Krebs cycle, gluconeogenesis, fatty acids, and protein biosynthesis^[^^6^^]^. 

PCD (MIM#266150) is a rare metabolic disorder with a recessive mode of inheritance due to biallelic mutations in the *PC* gene. PCD manifests in three primary clinical forms: A or infantile (North American) type, B or neonatal (French) type, and C or benign type. Subtype B is the most severe form and presents in the first hours of birth, while subtype A becomes apparent several months after birth. Of note, subtype C is the mildest and late-onset form. In other words, types A and B are both early-onset, and almost all affected individuals die in infancy or early childhood^[^^7^^,^^8^^]^. In type B, the level of consciousness and neurological status are typical at birth. Nevertheless, following a metabolic derangement with hyperammonemia, hypoglycemia, hyper-citrullinemia, and lactic acidosis in the first hours, a hypokinetic‐rigid syndrome occurrs. This condition is associated with hypotonia, abnormal ocular movements, tremor of the limbs, and early death. In some patients, seizure and liver insufficiency develop in the first months of life, and patients die within a few months^[^^9^^,^^10^^]^. However, type A presents several months after birth with increasing hypotonia and generalized developmental delay, failure to thrive, profound apathy, and death in infancy or early childhood. In addition, patients may show intermittent lactic acidosis, especially during infections and other physiologic stresses^[^^6^^,^^8^^]^. On the other hands, type C is a late-onset and benign form that presents with recurrent episodes of ketoacidosis, mild psychological delay, and neurologic symptoms in infancy or early childhood^[^^7^^,^^8^^]^. 

This study aimed to illustrate the genetic cause of metabolic disorders and early death in two newborns admitted to the NICU for a metabolic decompensation. WES was carried out for the patients, and two novel variants were identified in the *PC* gene, one of which was classified as likely pathogenic and the other as VUS, according to the ACMG guidelines. The wild-type protein and the identified variants were modeled, and the effect of the variants on their secondary and tertiary structures was assessed. Moreover, the free energy of the normal protein and its interactions with ligands were scrutinized and compared with the novel (p.R156P) and a previously reported (p.R156Q) variant. Finally, the results of the evaluations provided evidence of pathogenicity for both identified variants, causing the early-onset form (type B PCD).

## MATERIALS AND METHODS

**Individuals**

Two neonates (0003 and 0012 from the family No. 0003 and 0012), who were suspected of inherited metabolic disorders and admitted to the NICU of Ali-Asghar Pediatric Hospital, Tehran, Iran, were chosen for the study. Blood samples (5 ml) were taken from the neonates and available parents after performing pre-test genetic counseling. 

**Sample collection and DNA extraction**

 DNA was extracted from blood samples using the QIAamp DNA Blood Mini Kit (Qiagen, Hilden, Germany) according to the manufacturer’s instructions. The quantity and quality of DNA samples were evaluated by Thermo Scientific™ NanoDrop™ (1.8>A280/A260>2; Thermo Scientific, Waltham, MA, USA) and run on an agarose gel.

**Whole-exome sequencing**

According to the clinical evaluations and initial biochemical tests, all the patients were suspected of an inherited metabolic disorder; hence, in addition to essential therapeutic approaches, WES was performed to accurately diagnose the genetic diseases. For this purpose, genomic DNA was fragmented, and all exons of protein-coding genes and flanking sequences were enriched using SureSelect Human All Exon V6 Kit (Agilent Technologies, Santa Clara, CA, USA). Subsequently, the generated library was sequenced on the NextSeq 500 platform (Illumina, San Diego, CA, USA) with an average coverage depth of 100×. After base calling, trimming the adapters, and FASTQ file quality controls, low-quality reads were eliminated. Next, reads were aligned to the reference human genome (hg19) using the Burrows-Wheeler Alignment tool^[^^11^^]^, and duplicates were removed via Picard tools (http://broadinstitute.github.io/picard). Subsequently, variant calling was performed by the HaplotypeCaller tool, from Genome Analysis Toolkit version 4.0 (GATK4) package^[^^12^^]^. Annotation was then conducted by ANNOVAR^[^^13^^]^ (https://wannovar.wglab.org), which extracts allele frequencies from known population databases, including the 1000 Genomes project (1000G, http://www.internationalgenome.org)^[^^14^^]^, the exome aggregation consortium (ExAC, https://exac. broadinstitute.org/)^[15]^, genome aggregation database (gnomAD, https://gnomad.broadinstitute.org/)^[^^16^^]^, and Kaviar dataset (https://db.systemsbiology.net/ kaviar/)^[^^17^^]^. To predict pathogenicity and the potential effect of variants on protein structure and function as follows, we employed ANNOVAR using the datasets: SIFT (https://sift.bii.a-star.edu.sg/)^[^^18^^]^, PolyPhen-2 (http://genetics.bwh.harvard.edu/pph2/)^[^^19^^]^, Mutation Taster (http://www. mutationtaster.org/)^[^^20^^]^, PANTHER (http://www.pantherdb.org/)^[^^21^^]^, and PROVEAN (http:// provean.jcvi.org/)^[^^22^^]^. After analyzing WES data, we used sequential filtering to select the most relevant variants. Then, considering the clinical symptoms, the following databases were used to exclude irrelevant variants: Online Mendelian Inheritance in Man (OMIM) (https://www.omim.org/), GeneReviews® (https:// www.ncbi.nlm.nih.gov/books/NBK1116/), and Gene Cards (https://www.genecards. org/). At the end, the pathogenicity of the final variants was evaluated in ClinVar (https://www.ncbi.nlm.nih. gov/clinvar/), dbSNP (https://www.ncbi.nlm.nih. gov/snp/), InterVar (http://wintervar.wglab.org/), and Varsome (https:// varsome.com/variant/hg19/)^[^^23^^]^ datasets. Besides, to assess the allele frequency of the final variants in Iranian ethnicity, Iranome (http://www.iranome.com) database was utilized.

**Confirmatory analysis by Sanger sequencing**

Variants identified in the neonates and the available parents were confirmed through Sanger sequencing. Primer sequences are presented in Supplementary Table 1. Finally, the pathogenicity of the variants was evaluated according to the ACMG guidelines^[^^24^^]^.

**Modeling and protein structure analysis**

***Sequence and structure of proteins***

The PC sequence was retrieved from the UniprotKB database (ID: P11498; https://www.uniprot.org/ uniprotkb/) and submitted to SWISS-MODEL for model building. The homology modeling pipeline identifies target-template proteins based on BLAST (https://blast.ncbi.nlm.nih.gov/Blast.cgi) and HHblits (https://toolkit.tuebingen.mpg.de/tools/hhblits). The resulting models were ranked based on GMQE, a dataset indicating the quality of the models^[^^25^^]^. In addition, homology modeling for the frameshift variant c.908delG:p.G303Afs*40 was performed by the I-TASSER server (https://zhanggroup.org/I-TASSER/) for subsequent comparison with wild-type PC.

***Evaluation of the SNP effects on the protein secondary and tertiary structure***

The PSIPRED server (http://bioinf.cs.ucl.ac.uk/ psipred/) was utilized to predict the secondary structure of wild-type and mutant (R156Q and R156P) proteins^[^^26^^,^^27^^]^. Furthermore, 3D structures of the mutant proteins were generated using Chimera (version 1.8)^[^^28^^]^. To remove atomic clashes of the protein structure, energy minimization was implemented in 100 steps (step size = 0.02 Å) using AMBER f12SB force field parameters and the steepest descent and conjugate gradient algorithms. The functional form of the PC enzyme is known as a homotetramer that interacts with the ligands ATP, biotin, and pyruvate. Hence, in the wild-type and PC variants modeling, the studied monomer format was in complex with ATP. This behavior was due to the position of mutations R156P and R156Q in the ATP binding domain. Finally, the binding affinity of proteins and ligands was calculated and compared using the PRODIGY server (https:// wenmr.science.uu.nl/prodigy/)^[^^29^^]^. 

***Prediction of protein structure flexibility***

Amino acid fluctuations were predicted during coarse-grained simulation using the CABS-flex web server (http://biocomp.chem.uw.edu.pl/CABSflex/)^[^^30^^]^. The residue fluctuation profile was generated as an interactive 2D plot displaying the RMSF of the amino acids during the simulation. Protein structures were uploaded as PDB format files, and the simulations were performed with default parameters.

***Prediction of functional SNPs effect by PolyPhen-2***

PolyPhen-2 estimates the possible effect of residue replacement using supervised machine learning. It predicts the probability for a mutation to be damaged based on the information of the protein sequence, phylogenetics, and structure. PolyPhen-2 uses datasets HumDiv and HumVar from the UniProtKB database for training and testing the predicted models. HumDiv contains collections of human Mendelian disease-causing mutations and differences between homologous human and mammalian proteins with non-deleterious effects, while HumVar includes all pathogenic and non-pathogenic human SNPs^[^^19^^]^.

***Prediction of the functional SNP effect by SNAP2***

SNAP2 (https://rostlab.org/services/snap2web/) server was used to evaluate the functional outcome of R156 substitutions with either P or Q on PC protein. The SNAP2 score was calculated according to the data of multiple sequence alignments, secondary structure, and solvent accessibility of the protein sequence using the neural network method^[^^31^^]^. 

***Prediction of the functional consequence of SNPs by SNPs&GO***

SNPs&GO (http://snps.biofold.org/snps-and-go) predicts whether the single amino acid substitution affects the function of a protein. Using a sequence-based algorithm, SNPs&GO calculates the pair-wise alignments with the BLAST algorithm against the UniRef90 dataset to generate the sequence profile. The probability for mutations to be damaging is calculated using sequence feature data, frequency data of different residues as the sequence environment, and the functional annotation score related to gene ontology terms. However, in the structure-based algorithm, SNPs&GO uses the structural environment and relative solvent-accessible region data for the wild-type amino acid^[^^32^^]^. 

***Prediction of protein stability by I-Mutant 2.0***

The PC stability changes were predicted based on the protein sequence and structure using I-Mutant 2.0 server acting based on a support vector machine tool^[^^33^^]^. The impact of mutation was evaluated by calculating the chanhe in the free energy value (ΔΔG) of the protein. A positive ΔΔG value indicates increased stability of the protein, and a ΔΔG more than 0.5 suggests significant stability; however, a negative ΔΔG value represents decreased stability. The values of the reliability index ranged from 0 to 10.

***Prediction of functional, molecular, and phenotypic effects of SNPs by FATHMM***

FATHMM server is a sequence evolutionary-based tool that predicts the functional influence of SNVs on protein. The computational prediction uses Hidden Markov Models to evaluate the sequence conservations by aligning homologous sequences in an unweighted/ species-independent method^[^^34^^]^. A negative score indicates the probable damaging effects of an amino acid substitution.

## RESULTS

**Clinical features of the disease**

The probands (neonates 0003 and 0012) were the results of consanguineous marriages with no family history of similar conditions. In both neonates, the disease started at the first day of the life, and they deceased in a couple of days. The results of first level-biochemical tests demonstrated hypoglycemia, hyperammonemia, lactic acidosis, and disturbed LFT tests in both patients, suggesting a metabolic disorder. However, no definitive diagnosis was possible, and the patients died before the diagnostic workup was completed.

**WES and mutation detection**

**Fig. 1:**
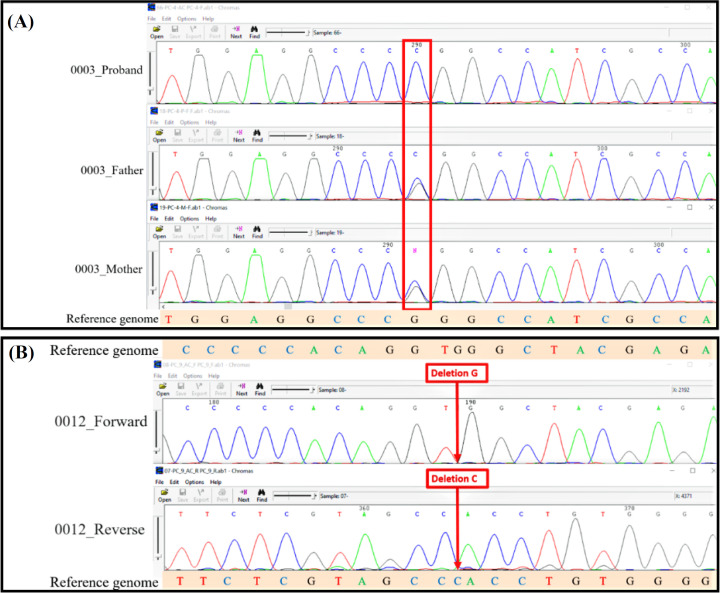
Sanger sequencing electrophorograms. (A) Sanger sequencing confirmed the homozygous status of the proband 0003 (above) for the missense variant of c.467G>C and the heterozygous state of his father (middle) and mother (below); (B) Sanger sequencing results confirmed the homozygous c.908del frameshift variant in proband 0012 with the input sequences

In patient 0003, WES data analysis revealed a novel homozygous missense variant, c.467G>C, p.R156P, NM_000920.4, in exon 5 of the *PC* gene related to PCD (MIM#266150). Sanger sequencing successfully confirmed the genotypes in the proband and his parents (Fig. 1A). According to the ACMG guidelines, this variant was categorized as a VUS^[^^23^^,^^24^^]^. In patient 0012, the novel homozygous frameshift variant, c.908del, p.G303Afs*40, NM_000920.4, was detected in exon 9 of the *PC* gene, which was confirmed by Sanger sequencing (Fig. 1B). However, it was impossible to confirm the parents’ heterozygous genotype due to the unavailability of blood samples. In accordance with the ACMG guidelines, this variant was categorized as likely pathogenic^[^^23^^,^^24^^]^. Given the recent recommendation about PP3^[^^35^^-^^37^^]^, the variant c.467G>C, p.R156P, having PP3, PM2, and PM5 criteria, and the variant c.908del, p.G303Afs*40, with PVS1, PM2, and PP5 criteria, are classified as likely pathogenic, and pathogenic variants, respectively. In addition, according to the patient’s phenotype, clinical presentation, and parental consanguinity, both variants get PP4 criterion in both patients.

**Homology modeling and protein structure analysis**

***Modeling the protein 3D structure***

**Fig. 2:**
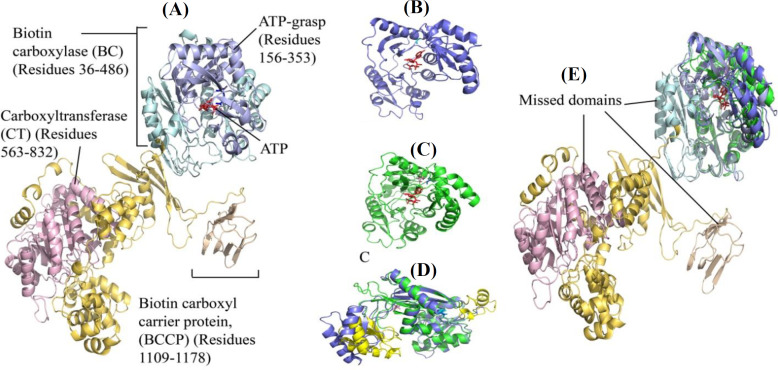
Cartoon view of PC protein and PC frameshift variant models. (A) Domains of PC protein by focusing on ATP-grasp domain; PC frameshift variant modeled by SWISS-MODEL(B) and I-TASSER (C); (D) Structural alignment of PC frameshift variant models where the differences are yellow colored; (E) Structural alignment of PC frameshift variants models and wild-type PC showing missing domains

The canonical isoform of PC and its functional domains are depicted in Figure 2A. The crystal structure of *Staphylococcus aureus* PC (3BG5) was proposed as a template for modeling. This template had 48.86% sequence identity and 43% sequence similarity with human PC^[^^38^^]^. The GMQE score between 0 and 1 indicates an overall quality estimation based on the template coverage (Table 1). The crystal structure of the biotin carboxylase PC (2DZD) domain was used for modeling the frameshift mutant by SWISS-MODEL, where it shares 58.06% and 47% sequence identity and similarity, respectively, with input sequence^[^^39^^]^. The crystal structure of biotin carboxylase from *Staphylococcus aureus* (2VPQ) was the top template proposed by I-TASSER to model the PC frameshift variant^[^^40^^]^. In the I-TASSER output, the models are ranked by c-score, which indicates the overall quality of the predicted models, from -5 to 2. Higher values in c-score indicate higher modeling accuracy. Comparing two proposed structures for the PC frameshift mutation indicated the complete coverage of the I-TASSER model with 341 amino acids, while SWISS-MODEL presented a 276-amino acid model for residues 34-310 (Fig. 2B and 2C). Alignment of the two models showed RMSD of 0.721Å. Differences in the models in terms of 3D conformation are in yellow (Fig. 2D). The missing domains of the PC frameshift variant models compared to the wild-type PC are depicted in Figure 2E.

***SNV effects on secondary and tertiary structure of protein***

**Fig. 3:**
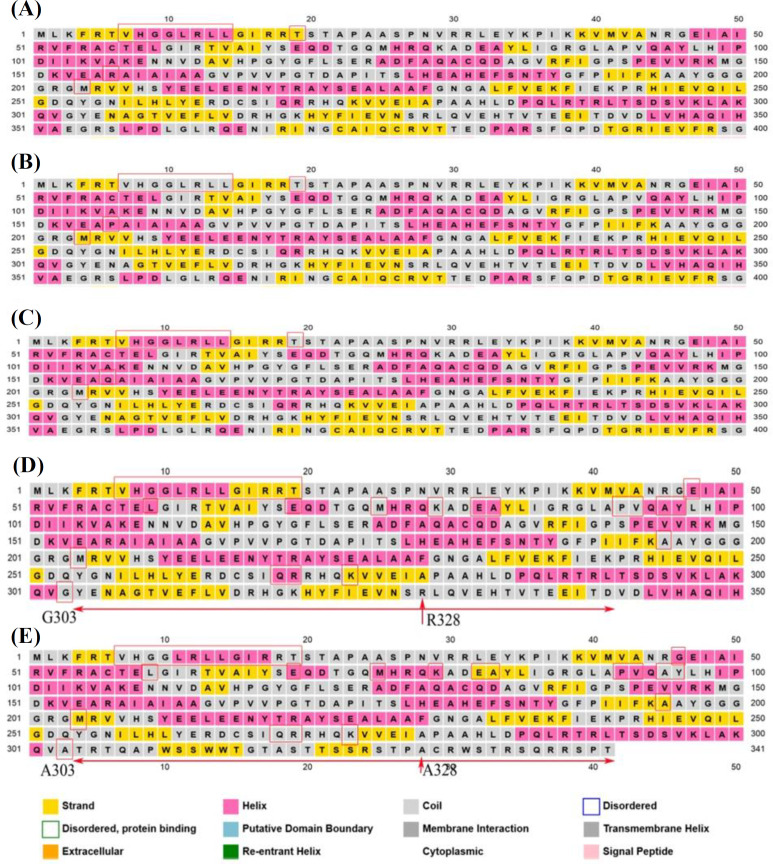
The secondary structure prediction of wild-type PC and variants. (A) Wild-type PC protein (residues 1-400); (B) PC-R156P protein; (C) PC-R156Q protein; (D) Wild-type PC protein (residues 1-350); (E) PC frameshift variant

**Fig. 4:**
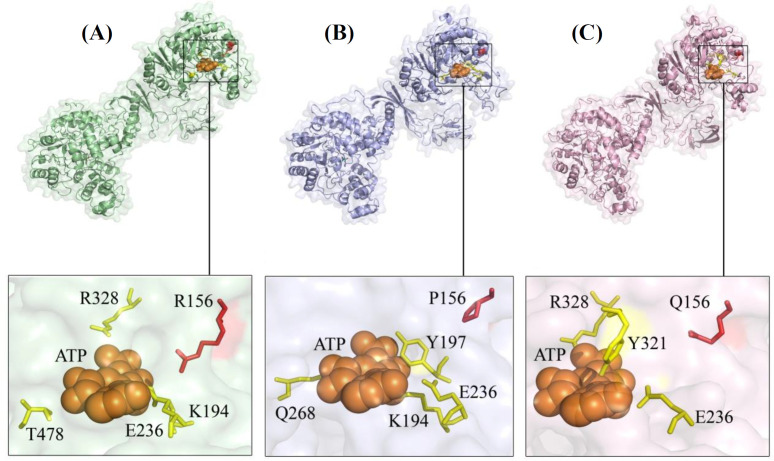
The tertiary structure of PC protein and its variants complex with ATP. (A) Wild-type PC protein and residues involved in the interaction with ATP; R156P (B) and R156Q (C) proteins and residues involved in the interaction with ATP

The secondary structure of wild-type PC and its variants was evaluated by PSIPRED. Although only one residue was changed, slight differences were predicted for the 2D form of the R156P variant. However, the secondary structure around the substituted residues, either P156 or Q156, showed no variation compared to the wild-type residue (R156). Differences in the predicted secondary structure of wild-type PC and variants (residues 1-400) are designated in Figure 3A, 3B, and 3C. Secondary structure prediction of the G303A PC frameshift mutant indicated several variations compared to the wild-type PC. The most significant difference was observed in the protein sequence after the mutation site G303A, where the sequence was completely changed. This includes the R328, a critical amino acid in ATP binding, which was replaced by alanine (Fig. 3D and 3E). Since the R156P and R156Q mutations are located in the ATP-grasp domains of PC, the effect of SNVs on the 3D structure was evaluated based on the interactions of the proteins with ATP ligand. The wild-type PC and ATP interaction pattern indicated residues K194, E236, R328, and T478. Residue profile in PC-R156Q and ATP interaction was composed of E236, R328, and Y321, while in PC- R156P consisted of K194, Y197, E236, and Q268 (Table 2). The binding affinity of proteins and ATP indicated a higher tendency of wild-type PC to ATP than the variants (Fig. 4).

***Generation of residue fluctuation profile***

**Fig. 5:**
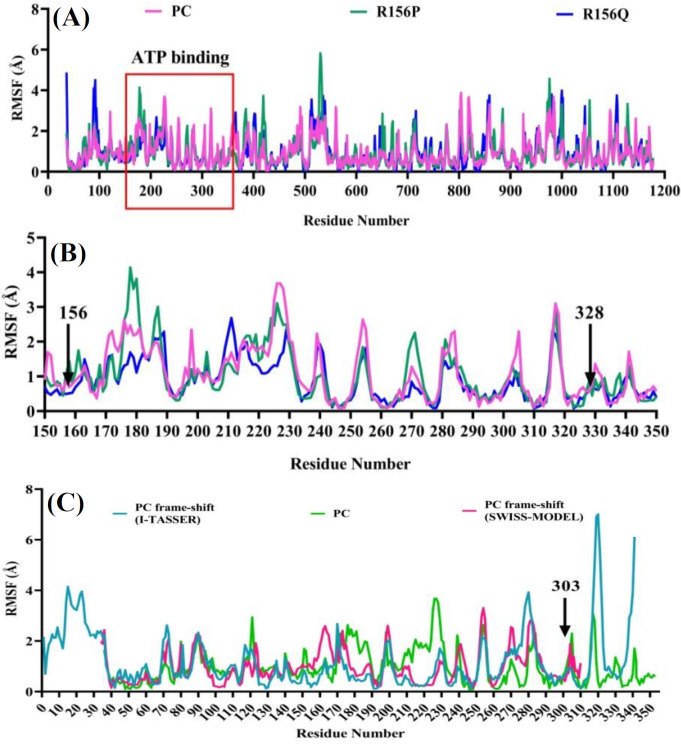
RMSF plot of residues during the simulation. (A) PC protein and its variants (R156P and R156Q); (B) Close-up view of fluctuations in the ATP-binding domain (~150-350); (C) RMSF plot of PC frameshift variant models by I-TASSER and SWISS-MODEL compared to PC protein

Residue fluctuation profiles were generated as the RMSF plots of amino acids during the simulations of wild-type and variants PCs (Fig. 5A and 5B). A close-up view of the fluctuations in the ATP-binding domain (residues 150-350) is displayed in Figure 5B. In spite of no significant change in the fluctuations of residues 156 and 328, which are critical in ATP binding, other residues in the ATP binding domains displayed variations, showing different affinities to ATP. On the other hand, residues in both frameshift variant models showed relatively similar fluctuations (Fig. 5C). Since the sequence of PC frameshift has changed from residue 303, a different fluctuation pattern was detected at the C-terminal compared to the wild-type PC protein.

***Analysis of SNP effects by Polyphen-2 and SNAP2***

**Fig. 6:**
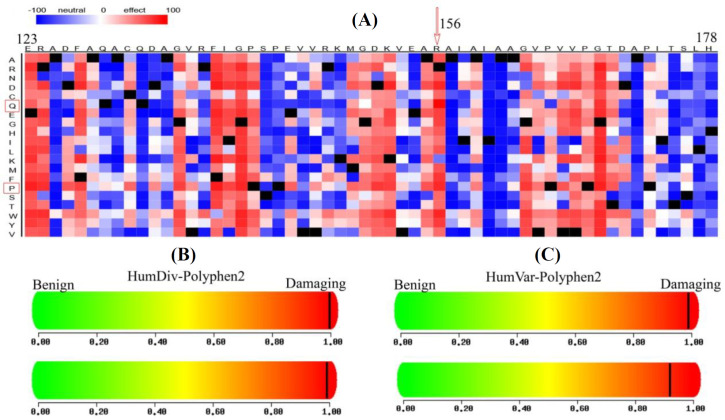
Prediction of SNP effect on PC protein. (A) Heatmap by SNAP2 (residues 123-178); HumDiv (B) and HumVar (C) results for R156P (above) and R156Q (below) substitutions

The SNAP2 server predicted SNVs R156P and R156Q as functionally effective mutations with 91% and 95% accuracy and scores of 84 and 97, respectively (Table 3). The scores ranged from -100 to + 100, showing “strong neutral” and “strong effect” on protein function, respectively. A heatmap representation of residues 123-178 is depicted in Figure 6A, indicating the high impact of R156 substitutions. Polyphen-2 scores predicted the R156P and R156Q variants as “damaging” (Table 3). Likewise, the HumDiv and HumVar indicators are located in the red zone (Fig. 6B and 6C), indicating both variants were damaging.

**Table 1:** Homology modeling of proteins using SWISS-MODEL and I-TASSER

**Protein**	**Template ** **PDB ID**	**Sequence ** **identity (%)**	**Sequence ** **similarity (%)**	**Coverage**	**GMQE**	**C-score**
PC	3BG5	48.86	43	0.96 (36-1178)	0.71	-
PC^*^ (frameshift mutation)	2DZD	58.06	47	0.84 (34-310)	0.70	-
PC^**^ (frameshift mutation)	2VPQ	48	-	0.87 (37-337)	-	-0.06

^ *^modelled by SWISS-MODEL; ^**^modelled by I-TASSER

**Table 2:** Evaluation of SNP effects on binding affinity and interactions

**Protein**	**ΔG**	**ATP residues in interaction**
Wild-type PC	-7.6	K194, E236, R328, T478
PC-R156Q	-7.2	E236, R328, Y321
PC-R156P	-7.1	K194, Y197, E236, Q268

***Prediction of mutation effect using I-Mutant 2.0, FATHMM, and SNPs&GO servers***

PC stability changes due to the SNVs were evaluated by I-Mutant 2.0 according to the protein sequence and structure, which are summarized in Table 3. Since negative ΔΔG scores represent decreased protein stability, the data revealed a destabilizing effect of both variants on PC protein. Furthermore, SNP analysis by FATHMM calculated negative scores (-4.59 and -4.57 for R156P and R156Q, respectively), indicating a damaging effect of R156P and R156Q on PC protein. In addition, the SNPs&GO predicted both mutations as disease-causing with a probability greater than 0.5 (Table 3).

**Table 3:** Evaluation of SNP effects on PC protein

**Variant**	**Polyphen-2**				**SNAP2**			**SNPs&GO (sequence)**		
	**Score**		**Prediction**		**Score**	**Prediction**		**Probability**	**Prediction**	**RI**
	**HumDiv**	**HumVar**								
R156Q	0.991	0.919	Damaging		97	Effect		0.639	Disease	3
R156P	0.995	0.998	Damaging		84	Effect		0.893	Disease	8
					
**Variant**	**I-Mutant**				**FATHMM**			**SNPs&GO**		
	**ΔΔG (kcal/mol)** **(sequence)**	**Stability prediction**	**ΔΔG (kcal/mol)** **(structure)**		**Score**	**Prediction**		**Probability**	**Prediction**	**RI**
R156Q	-0.7	Decrease	-1.29		-4.57	Damaging		0.896	Disease	8
R156P	-0.55	Decrease	-1.28		-4.59	Damaging		0.956	Disease	9

RI, reliability index

## DISCUSSION

**Fig. 7:**
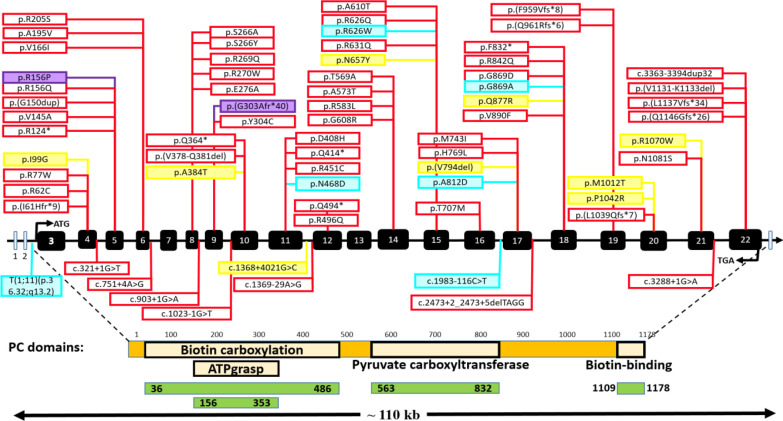
**Review of the reported** ***PC*** **gene variants. E** **xonic and intronic variants** **are** **shown above and below the** ***PC*** **gene, respectively.** **Red boxes: deleterious variants publishe** **d** **in HGMD professional (ver.2022.2) (https://www.hgmd.cf.ac.uk/ac/gene.php?gene=PC); yellow boxes: variants of uncertain significance, according to HGMD (ver.2022.2); blue boxes: novel variants reported by Tsygankova et al.** ^[^^41^^]^ **; violet boxes: suggested variants in the present study**

In the present study, WES revealed that patients 0003 and 0012 were homozygous for the novel variants c.467G>C (p.R156P) in exon 5 and c.908del (p.G303Afs*40) in exon 9 of the *PC* gene, respectively. Both variants were confirmed by Sanger sequencing in the probands and available parents. According to the ACMG guidelines^[^^24^^]^ and recent statement about PP3 criteria^[^^35^^-^^37^^]^, the variant in patient 0003, c.467G>C (p.R156P), is likely pathogenic, and that in patient 0012, c.908del (p.G303Afs*40), is a pathogenic variant. In addition, based on the patient’s clinical presentation and parental consanguinity, both variants received an additional PP4 score. Interestingly, Wang et al*.*^[^^8^^]^ reported another variant, c.467G>A (p.R156Q), at position 156, which has been reported to be a VUS in ClinVar^[^^8^^]^, and seems to be likely pathogenic in VarSome (2022)^[^^23^^]^, and pathogenic in UniProt^[^^7^^]^. In patient 0012, the frameshift variant c.908del induced an aberrant stop codon after the addition of 40 amino acids and activation of the nonsense-mediated mRNA decay mechanism. This variant changes the protein active site and eliminates the crucial functional domains, such as pyruvate carboxyltransferase and biotin-binding domains, as well as disrupts the biotin carboxylation and ATP grasp domains (Fig. 7). 

Currently, 62 disease-causing variants, including 42 missense/nonsense, 7 splicings, 7 small deletions, 5 small insertions, and 1 minor duplication, have been reported for the *PC* gene in HGMD Professional 2021.4 (https://www.hgmd.cf.ac.uk/ac/ gene.php?gene=PC). Tsygankova et al.^[^^41^^] ^have reported four exonic variants, one chromosomal translocation, and one deep intronic variant related to PCD. The present study suggests two additional pathogenic variants, c.467G>C and c.908del, to expand the* PC *variants spectrum (Fig. 7).

Surprisingly, few variants have been reported in this gene, which might be due to the early-onset and lethal nature of forms A and B of the PCD^[^^7^^,^^8^^]^. 

PCD (MIM#266150) is a rare autosomal recessive disorder resulting from biallelic mutations in the *PC* gene (https://www.ncbi.nlm.nih.gov/gene/5091). The high frequency of consanguineous marriages in countries like Iran can increase the prevalence of rare autosomal recessive disorders^[^^42^^-^^44^^]^. However, there are few reports of PCD in the Iranian population. Bayat et al.^[^^45^^]^ have reported a two-year-old girl from Iran with c.806G>A variant in the *PC* gene and a longer survival^[^^45^^]^. All patients in the present and previous studies were resulted from consanguinous marriages. PCD manifests in three primary clinical forms, A, B, and C. Unlike early onset types A and B, patients with type C often survive until late adolescence^[^^8^^]^. According to the biochemical test results, clinical course, and disease survival, the patients in this study seem to be affected by type B PCD. PC enzyme activity in fibroblasts culture is a well-known indicator for PCD diagnosis in newly diagnosed patients^[^^8^^]^, though it cannot indicate the extent of PC activity impairment^[^^3^^]^. However, PC enzyme assay and fibroblast culture are inaccessible in Iran. 

An earlier study has indicated that pathogenic missense variants typically belong to the 2/3 N‐terminal end of the PC. On the other hand, mutations in the 1/3 C‐terminal end are tolerable and unlikely to cause disease^[^^7^^]^. Furthermore, it has been reported that at least one truncating variant leads to type B and a neonatal-fatal form of PCD, while homozygous missense variants are the genetic cause of type A PCD^[^^7^^, ^^10^^]^. In complete agreement with Monnot et al.’s^[^^7^^]^ and Carbone et al.’s^[^^10^^]^ investigations, our study revealed that the patient 0012 was homozygous for the truncating variant c.908del, p.G303Afs*40 related to type B PCD. However, the causative mutations of all type B patients are not nonsense; there are reports of biallelic missense variants in patients presenting type B PCD^[^^8^^,^^46^^,^^47^^]^. Likewise, one of the patients in this study who expired from type B PCD in the neonatal period, inherited the homozygous missense variant c.467G>C, p p.R156P. However, Monnot et al.^[^^7^^]^ reported the variant p.R156Q for the first time as a cause of type A PCD. Nevertheless, the genotype/phenotype correlations in type A and C PCD have not fully been elucidated, since both types have been observed with biallelic missense mutations^[^^3^^]^. Recently, in silico free energy calculations have increasingly been used to evaluate the effect of residue substitutions on protein structure, stability, and ligand binding affinity^[^^43^^,^^48^^-^^52^^]^. In 2019, Coci et al.^[^^3^^]^ estimated the stability of variant PCs by calculating the double free energy difference (∆∆G). They reported that p.R156 is a residue close to the ATP binding site, and the variant p.R156Q in the protein biotin carboxylation domain destabilizes the PC structure. In complete accordance with Coci et al*.*^[^^3^^]^, our free energy calculations exhibited a destabilized PC due to p.R156Q and p.R156P. Moreover, protein and ligand binding affinity evaluations indicated a higher affinity of wild-type PC to ATP than their variants (Fig. 4). 

WES data analysis, confirmatory Sanger sequencing, pathogenicity evaluations according to ACMG guidelines, and in silico protein assessments revealed a novel missense variant, c.467G>C, p.R156P, in patient 0003 and a novel frameshift variant, c.908del, p.G303Afs*40, in patient 0012, which both are suggested to be pathogenic relating to type B PCD. However, more studies with larger sample sizes and functional analysis are necessary to be carried out to confirm these observations. The results of this study could expand the variant spectrum for the *PC* gene and would be helpful for pediatricians, endocrinologists, geneticists, scientists, and the patients’ families. In addition, in the cases of VUS variants, homology modeling and in silico analysis of the effects of variants on protein stability and ligand interactions can improve the pathogenicity score of the variants.

## DECLARATIONS

### Acknowledgments

The authors appreciate the patients’ families for their participation and the NICU staff (academic members, fellowships, residents, and nurses) of Ali-Asghar Pediatrics Hospital (Tehran, Iran) for their helpful cooperation. The authors also thank the employees of the Molecular Medicine Department, Pasteur Institute of Iran, Tehran, Iran, for their unique and friendly collaborations. 

### Ethical statement

The protocol of this study was authorized and approved by the Ethics Committee of the Pasteur Institute of Iran (ethical code: IR.PII.REC.1397.70). All the participants gave their informed consents.

### Data availability

For patients’ confidentiality reasons, data related to WES is available by reasonable requests from the corresponding author. 

### Author contributions

FM: wrote the concept of the study, designed the experiments, collected samples, performed the laboratory assessment, and analyzed and interpreted the data and prepared the initial draft of the paper; ER: performed the laboratory assessment and analyzed and interpreted the data and prepared the initial draft of the paper; EDD: revised the manuscript; NK: collected samples, wrote the concept of the study, and designed the experiments; ST: wrote the concept of the study; RM: provided materials and equipment and gave helpful advice at every stage; SZ: wrote the concept of the study, designed the experiments, and revised the manuscript. The study was carried out under the supervision of SZ and NK. All authors read and commented on the manuscript. 

### Conflict of interest

None declared.

### Funding/support

This work is a partial fulfillment of the Ph.D. thesis of Mrs. Fereshteh Maryami under supervision of Professor Sirous Zeinali and was financially supported by The Deputy of Research and Technology, Pasteur Institute of Iran, Tehran, Iran. 

## Supplementary materials

**Supplementary Table 1:** Primers sequences used for Sanger sequencing

**Gene**	**Transcript**	**Exon**	**Primers sequences**	**Tm (** **℃** **)**	**Length (bp)**
*PC*	NM_000920.4	Exon 5	5’ GACCAGGGAGTCTTAGTTGC 3’	61	612
			5’ CATCTTCACTCACCTCGTAGC 3’		
	NM_000920.4	Exon 9	5’ TGGAGCCTAGTGGACTACTT 3’	62	640
			5’ GCAAATGTCTCTGAGCCTTGC 3’		
